# Joint Hypermobility Syndrome in Patients With Functional Dyspepsia

**DOI:** 10.14309/ctg.0000000000000220

**Published:** 2020-11-04

**Authors:** Florencia Carbone, Asma Fikree, Qasim Aziz, Jan Tack

**Affiliations:** 1 Department of Gastroenterology, Neurogastroenterology & Motility, Translational Research in GastroIntestinal Disorders (TARGID), University of Leuven, Leuven, Belgium;; 2 Barts and The London School of Medicine and Dentistry, Wingate Institute of Neurogastroneterology, Centre for Neuroscience and Trauma, Blizard Institute Queen Mary University of London UK, London, United Kingdom.

## Abstract

**INTRODUCTION:**

The pathophysiology underlying functional dyspepsia (FD) is multifactorial and focuses on gastric sensorimotor dysfunction. Recent studies demonstrated that joint hypermobility syndrome (JHS) is strongly associated with unexplained dyspeptic symptoms in patients attending gastrointestinal clinics. We aimed to study the relationship between symptoms, gastric sensorimotor function, and JHS in FD patients.

**METHODS:**

Tertiary care FD patients who underwent a gastric barostat study and a gastric emptying breath test with 13C-octanoic acid were recruited for assessment of JHS. The presence of JHS was evaluated by a 2-phase interview and clinical examination that included major and minor criteria of the Brighton classification.

**RESULTS:**

A total of 62 FD patients (68% women, age 44 ± 1.8 years, and body mass index: 21.7 ± 0.7 kg/m^2^) accepted to participate in the study. JHS was diagnosed in 55% of FD patients. Assessed symptom profiles during the visit did not differ between the groups. Delayed gastric emptying was not significantly more common in JHS group compared with non-JHS group (JHS group 32% vs non-JHS group 16%, *P* = 0.31). Prevalence of hypersensitivity to distention (JHS group 24% vs non-JHS group 29%, *P* = 0.76) and impaired gastric accommodation (JHS group 38% vs non-JHS group 42%, *P* = 0.79) was similar in patients with or without JHS. No correlations were found between the Beighton hypermobility score and gastric compliance (*r* = 0.09).

**DISCUSSION:**

A large subset of this study cohort of tertiary care FD patients has coexisting JHS. We did not identify any specific differences in gastric sensorimotor function between patients with and without JHS. Further prospective research will be required to elucidate the relationship between JHS, a multisystemic disorder with widespread manifestations, and FD symptoms.

## INTRODUCTION

Functional dyspepsia (FD) is defined (Rome IV criteria) by epigastric symptoms in the absence of any organic or metabolic disease that can explain the symptoms (1,2). FD is a prevalent functional gastrointestinal disorder, affecting 8%–12% of the general adult population (3,4) and 15%–20% of patients presenting to tertiary outpatient care (5–7). Pathophysiology of this condition is incompletely understood. Several studies have shown that FD is associated with gastric dysmotility such as impaired gastric accommodation (GA) and delayed gastric emptying (GE), gastric sensory dysfunction, impaired mucosal integrity and duodenal low-grade immune activation, and dysregulation of the gut–brain axis (8–13).

Recently, there has been increasing awareness of the coexistence of joint hypermobility syndrome (JHS) or, as recently defined, Ehlers–Danlos syndrome (hEDS): a heritable disorder of connective tissue, with gastrointestinal disorders, particularly functional gastrointestinal disorders (14–23). Joint hypermobility refers to increased passive or active movement of a joint beyond its normal range. JHS is a heritable noninflammatory connective tissue disorder with a reported prevalence of 20% that includes the movement of a joint beyond its normal range together with arthralgia of the joints (20,24). JHS is considered a widespread systemic disorder, involving not only musculoskeletal and cutaneous components but also the cardiovascular, gastrointestinal, visual, and neuromuscular systems (15,17,20).

Historically, several anatomical gastrointestinal abnormalities have been described in JHS, including hiatus hernias, rectal morphology abnormalities such as rectocele, diverticular disease, and visceroptosis of the bowel (24–26). More recent work has repeatedly shown that gastrointestinal symptoms such as reflux, constipation, bloating, and food intolerances are common in JHS; in fact, a high prevalence of irritable bowel syndrome and gastroesophageal reflux disease has been reported in observational studies (17,18,25,26). In a large cross-sectional study of patients attending gastrointestinal clinics, JHS was found to be strongly associated with dyspeptic symptoms, particularly meal-related symptoms such as postprandial fullness (20). Moreover, using logistic regression analysis, this association was interdependent on pain and autonomic measures, suggesting a possible role of hypersensitivity in the etiology of dyspepsia in these patients. In another study, patients with JHS had a high prevalence of motility problems in their gastrointestinal tract such as small bowel dysmotility, delayed GE, and delayed colonic transit (14). Both visceral hypersensitivity and gastric dysmotility are involved in the etiology of FD, but it is unknown whether they are also involved in the etiology of dyspeptic symptoms in JHS.

The aim of this study was to explore the relationship between JHS and FD symptoms and gastric sensorimotor function. The primary aim of this study was to quantify the JHS prevalence in a tertiary care FD patient cohort. The secondary aim was to characterize the gastric sensorimotor function in FD population with JHS (JHS group) in comparison with FD population without JHS (non - JHS group).

## METHODS

### Study design

The study was design using a retrospective recruitment approach. FD patients (aged 18–75 years) who underwent a full pathophysiological workup for evaluation of FD during 2006–2014 were contacted and invited to the hospital to assess their joint mobility status. During this visit, patients were asked to fill out the Rome III questionnaire to assess the frequency of their symptom in the past 6 months. Registration University Hospital of Leuven number reference: S56776. All authors had access to the study data and had reviewed and approved the final manuscript.

### JHS assessment

JHS was assessed using a structured interview and examination based on the Brighton criteria for the classification of JHS, which was revised in 1998 (25). Further details of the methods are described in the Supplementary Table and Supplementary Material (see Supplementary Digital Contents 1 and 2, http://links.lww.com/CTG/A427).

### GE breath test and gastric barostat study

The C13-breath test was used to measure GE rate. At the University Hospital of Leuven, the breath test for assessment of GE rate is considered a standard diagnostic tool. Details of these methods are described in the Supplementary Material (see Supplementary Digital Content 2, http://links.lww.com/CTG/A426 and http://links.lww.com/CTG/A427).

### Data analysis

Demographics and clinical characteristics were analyzed and compared between groups with nonparametric Mann-Whitney test. The findings on gastric sensorimotor function (gastric barostat and GE test results) in patients with and without JHS were compared by means of Student unpaired *t* test with nonparametric Mann-Whitney test; χ^2^ test was used to compare proportions. The relation between the total Beighton hypermobility score and the level of gastric compliance, sensitivity and GA, and GE rate were investigated with Spearman correlations. In all analysis, *P* < 0.05 was considered significant. All data are presented as mean ± SEM.

## RESULTS

### FD patient population

In total, 194 patients were contacted to participate in this study. Three of these patients were excluded due to age (>75 years), and 3 had deceased in the past years. Sixty-two FD patients (68% women, age 44 ± 1.8 years, and body mass index [BMI]: 21.7 ± 0.7 kg/m^2^) accepted to participate and presented to the University Hospital in Leuven (Belgium).

### JHS characterization and pathophysiological history

Based on the Brighton classification, JHS was diagnosed in 55% of FD patients. Other joint disorders were diagnosed in 6% of the patients (2 patients presented with lupus, 1 patient with ankylosing spondylitis and, 1 patient with rheumatoid arthritis). Finally, 39% of the patients did not present any joint disease or syndrome.

In the JHS group, the prevalence of female patients was significantly higher than that in the non-JHS group (JHS 74% vs non-JHS 63%, *P* = 0.02). Age and BMI parameters were similar in both groups (JHS group 43.4 ± 2.2 years and 21.9 ± 0.9 kg/m^2^; non-JHS group 43.9 ± 3.2 years and 22.4 ± 1.1 kg/m^2^, *P* = 0.89 and *P* = 0.46, respectively).

In agreement with the Brighton classification, patients with JHS showed greater percentages for high Beighton score. However, both populations showed signs of arthralgia. Patients with JHS also presented with more dislocations, subluxations, soft tissue lesions, abnormal skin (striae, hyperextensibility, thin skin, and papyraceous scarring), varicose veins, hernias (gastric hernias not included in the analysis), and prolapses (colon, uterine, or bladder). No differences were observed in abnormal skin and eye signs (drooping eyelids or myopia or antimongoloid slant) (Table 1).

**Table 1.**
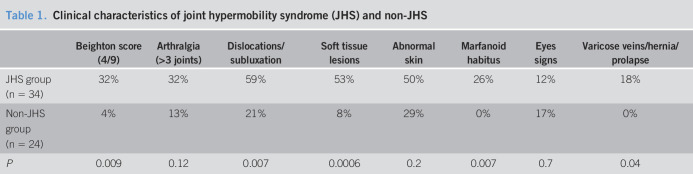
Clinical characteristics of joint hypermobility syndrome (JHS) and non-JHS

	Beighton score (4/9)	Arthralgia (>3 joints)	Dislocations/subluxation	Soft tissue lesions	Abnormal skin	Marfanoid habitus	Eyes signs	Varicose veins/hernia/prolapse
JHS group (n = 34)	32%	32%	59%	53%	50%	26%	12%	18%
Non-JHS group (n = 24)	4%	13%	21%	8%	29%	0%	17%	0%
*P*	0.009	0.12	0.007	0.0006	0.2	0.007	0.7	0.04

### Dyspepsia symptom characterization in JHS and non-JHS groups

Patients filled out the Rome III questionnaire during the visit. Assessed symptom profiles did not differ between the groups. Postprandial fullness (76% vs 82%, *P* = 0.88) and bloating (73% vs 77%, *P* = 0.94) were highly prevalent in both groups, followed by early satiation (58% vs 41%, *P* = 0.35), nausea (42% vs 36%, *P* = 0.86), belching (42% vs 36%, *P* = 0.44), and reflux (21% vs 5%, *P* = 0.18). Distribution of FD Rome III subgroups within the JHS and non-JHS groups was not different for postprandial distress syndrome (PDS) (21% vs 25%, *P* = 0.69), epigastric pain syndrome (12% vs 17%, *P* = 0.59), and the overlap PDS-epigastric pain syndrome (68% vs 58%, *P* = 0.47) subgroups.

### Gastric emptying

Data for GE were available for 81% of FD patients. Of these patients, 74% had a normal GE rate (T_1/2_ = 66.9 ± 3.3 minutes) and 26% had delayed (T_1/2_ = 156.2 ± 17.0 minutes). GE rate was assessed in 28 patients who showed JHS characteristics (82% women, age 43.9 ± 2.3 years, and BMI 22.8 ± 1.0 kg/m^2^) and in 19 patients without JHS (63% women, age 44.4 ± 3.8 years, and BMI 22.2 ± 1.2 kg/m^2^). No significant difference in average half emptying time was observed between both groups (average GE T_½_ in JHS group 95.0 ± 9.9 minutes vs average GE T_½_ in non-JHS group 82.0 ± 11.6 minutes, *P* = 0.31). Delayed GE was not significantly more common in JHS group compared with non-JHS group (JHS group 32% [n = 9/19] vs non-JHS group 16% [n = 3/16], *P* = 0.31; odds ratio = 0.39).

### The gastric barostat

All patients underwent a gastric barostat test. Impaired GA was found in 38% of patients, and 26% were hypersensitive to gastric distention, 8% of which showed both abnormalities, and 45% showed normal gastric barostat results. In the JHS and non-JHS groups, the minimal distention pressure was similar (6.7 ± 0.4 vs 5.8 ± 0.4 mm Hg, respectively, *P* = 0.15).

**Figure 1.**
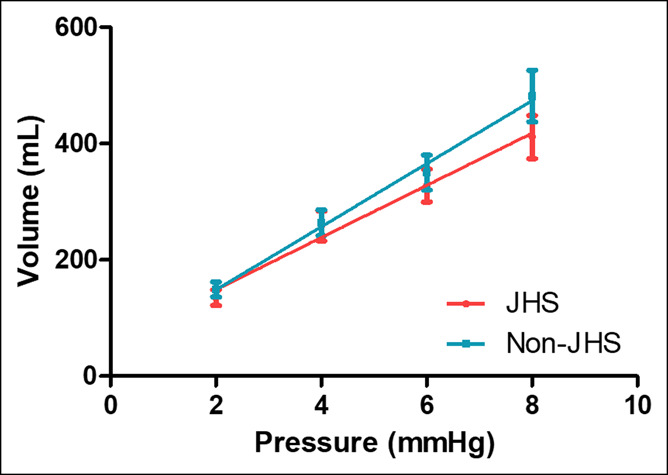
Gastric compliance of patients with functional dyspepsia with joint hypermobility syndrome (JHS) and without JHS. Representation of average intragastric balloon volume per increasing intragastric balloon pressure. No significant difference was observed in gastric compliance (slope volume/pressure) between groups (*P* = 0.60).

The gastric compliance of the JHS group was not significantly different to that of the non-JHS group (JHS group 60.2 ± 5.4 mL/mm Hg vs non-JHS group 54.1 ± 4.5 mL/mm Hg, *P* = 0.60). No differences were observed at sensory threshold volumes (JHS group 176.6 ± 23.3 mL vs non-JHS group 188.3 ± 22.2 mL, *P* = 0.32) and discomfort volumes (JHS group 483.9 ± 35.3 mL vs non-JHS group 530.7 ± 44.0 mL, *P* = 0.39) (Figure 1). Hypersensitivity to gastric distention in patients with JHS was similar to that of the patients without JHS (JHS group 24% [n = 8/34] vs non-JHS group 29% [n = 7/24], *P* = 0.76; odds ratio = 0.74).

**Figure 2.**
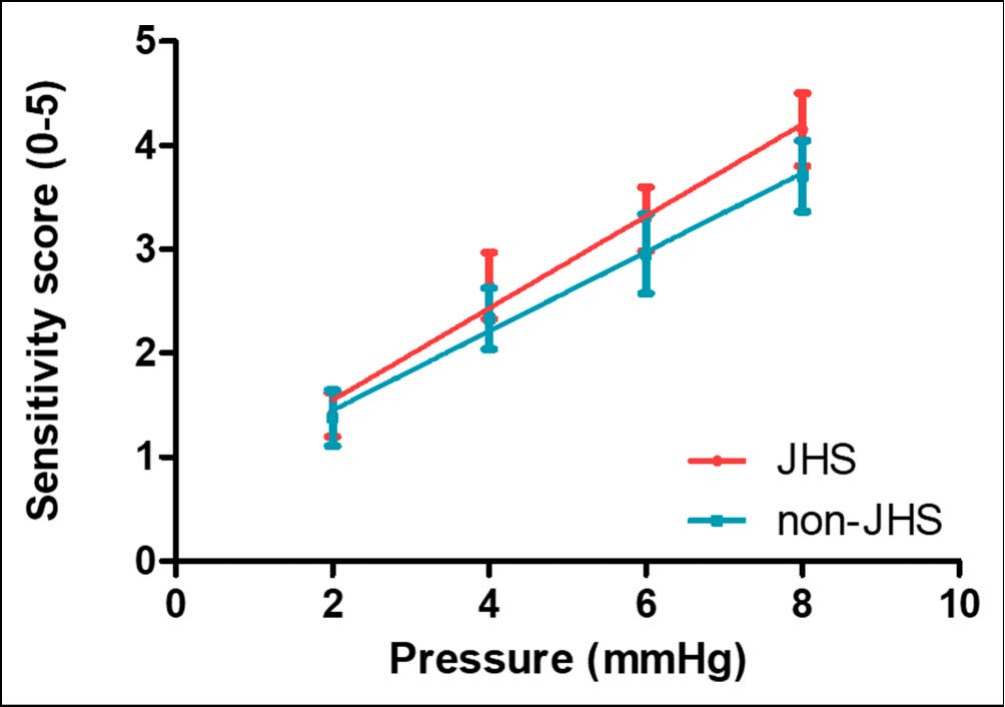
Gastric sensitivity to distention of patients with functional dyspepsia with joint hypermobility syndrome (JHS) and without JHS. Increasing gastric perception per increasing intragastric balloon pressure. Patients with JHS did not display a significantly different sensitivity to gastric distention compared with patients without JHS (*P* = 0.53).

The average pressure at threshold sensation was 9.8 ± 0.6 mm Hg in the JHS group and 8.7 ± 0.6 mm Hg the in non-JHS group (*P* = 0.25). The average pressure at discomfort did not differ between the groups (JHS group 15.4 ± 0.7 mm Hg vs non-JHS group 15.5 ± 1.2 mm Hg, *P* = 0.64) (Figure 2).

**Figure 3.**
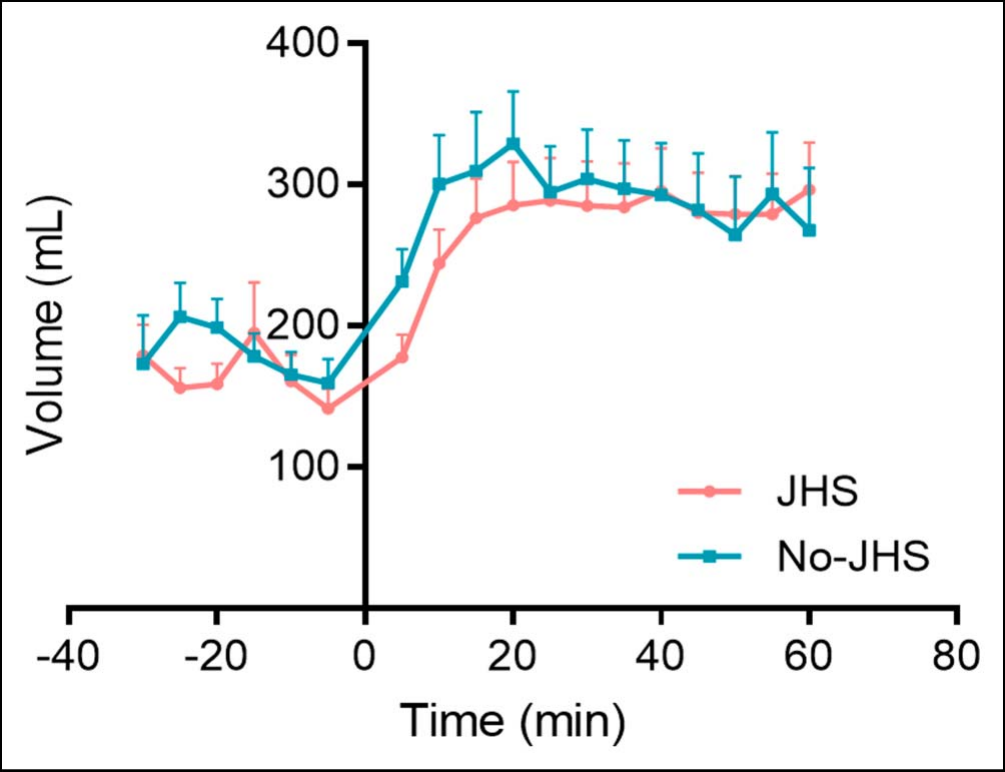
Gastric accommodation in patients with functional dyspepsia with joint hypermobility syndrome (JHS) and without JHS. Time 0 is the time in which the patients drank a nutrient drink (200 mL, 300 kcal). No differences in gastric accommodation were observed between the groups (*P* = 0.78).

The gastric sensitivity to distention (slope of sensitivity scores/mm Hg) showed no significant difference between the JHS and non-JHS groups (0.76 ± 0.1 mm Hg^−1^ vs 0.60 ± 0.06 mm Hg^−1^, *P* = 0.53) (Figure 3). Impaired GA was observed in 38% (n = 13/34) of patients with JHS and 42% (n = 10/24) of patients without JHS (odds ratio = 0.07; *P* = 0.79). The occurrence of both disorders, impaired GA and hypersensitivity to gastric distention, was observed in 9% of JHS group and 8% of non-JHS group. The meal-induced proximal stomach relaxation did not differ significantly between both groups (JHS group 99.2 ± 20.3 mL vs non-JHS group 112.4 ± 27.2; *P* = 0.78) (Figure 3).

### Occurrence of JHS and severity of gastric motility and sensitivity

No correlations were found between the Beighton hypermobility score and gastric compliance (*r* = 0.09, *P* = 0.54), gastric sensitivity (*r* = −0.21; *P* = 0.14), GA (*r* = −0.02; *P* = 0.91), or GE rate (*r* = 0.12 *P* = 0.43).

## DISCUSSION

In this study, we confirmed a high prevalence of JHS (55%) in FD patients. Compared with patients without JHS, those with comorbid JHS were characterized by a higher prevalence of female sex. The occurrence of PDS symptoms was in line with the frequency reported in previous studies (15,20). Furthermore, it has also been reported that patients with chronic uninvestigated dyspepsia have increased occurrence of joint and back pain (27), and therefore, it is conceivable that some of these patients might also have comorbid JHS.

Few data are available regarding gastric sensorimotor function in FD patients with JHS. In this study, the gastric barostat did not demonstrate differences in gastric compliance, sensitivity to gastric distention, or GA in patients with JHS compared with patients without JHS. In addition, the prevalence of delayed GE was similar in patients with and without JHS. This latter finding is not in line with a previous study that also used 13-C octanoic acid GE breath test in a cohort of 72 FD patients. This study showed an increased incidence of delayed GE in patients with JHS compared with patients without JHS (35% vs 11%, *P* < 0.05) (28). Differences in the methods used in the studies such as the selection of patients or the established cutoffs to define delayed GE could play a role in this inconsistency.

Gastric sensorimotor function was assessed by the gastric barostat, which might not be the ideal method to study subtle differences between patients with and without JHS. This technique is invasive and difficult to tolerate, and presence of the barostat bag might alter the intragastric distribution of a meal and exaggerate relaxation of the proximal stomach (29–31). Visceral sensitivity ratings are also not devoid of reporting bias through factors such as hypervigilance and anxiety (32). Nevertheless, the procedure is considered the gold standard to measure both sensitivity to gastric balloon distention and GA in FD (33–36).

In this study, we also observed, in addition to JHS, a very high prevalence of other joint disorders (up to 60% of patients were diagnosed with some kind of joint or rheumatological disorder), suggesting an important connection between gastrointestinal symptoms and rheumatological diagnoses. We identified patients with autoimmune diseases such as rheumatoid arthritis, lupus, and ankylosing spondylitis. Increased immune activation of mast cell or eosinophils has recently become an interesting topic related to FD (12,37–39). Furthermore, a higher risk of the diagnosis of FD has been observed in patients with rheumatological autoimmune disorders (40). The involvement of immunological factors in the development of FD suggests again an additional contributing factor to symptoms in at least some of the patients.

JHS is considered a multisystemic disorder with widespread manifestations (41), and even though we have not identified any differences between patients with and without JHS in specific gastric sensory motor function, we have, nevertheless, not examined the patients for other comorbidities such as postural tachycardia syndrome (POTS) (16,20,42,43), an autonomic disturbance, and mast cell activation syndrome (44), which have been associated with JHS and can cause gastrointestinal symptoms. Furthermore, an association among POTS, increased mast cell activation, and gastrointestinal symptoms has been observed, again suggesting an additional link to immunological factors (45,46). Given that these are treatable conditions, which can also lead to improvement in gastrointestinal symptoms, it is important to identify these patients in routine clinical practice to ensure that the comorbidities are suitably recognized and well managed.

Recently, deficiency of the extracellular matrix glycoprotein tenascin-X (TNX), encoded by the *TNXB* gene, has been classified as a specific subtype of hEDS (47). Patients with TNX deficiency have a phenotype that is very similar to that of JHS. In addition, TNX has been observed to be expressed in the gut tissue of men (stomach) and mice (colon and stomach) (23,48). Of interest, TNX has been shown to have a function in motility and to play an indirect role in visceral sensitivity, specifically for pain sensations. Again, in our study, we did not examine the TXN genotype in our patients, but these new insights highlights the importance to investigate in future studies the status of the *TXNB* gene in patients with functional gastrointestinal disorders.

Limitations in this study include the relatively small number of patients studied and the reliance on a historical barostat examination. However, at least in mid-term, barostat studies were found to be reproducible (49), but this has not been studied over several years. Even though the study is retrospective in nature for clinical evaluations and investigations performed, but, nevertheless, JHS is a trait, and this remains stable overtime. Hence, the analysis of FD features between patients with and without JHS remains valid. In addition, the study was performed in patients attending a tertiary care center specialized in functional gastrointestinal disorders. FD patients were diagnosed as defined by the Rome III criteria by a specialized gastroenterologist (J.T.); all patients had chronic dyspeptic symptoms for at least 6 months, an upper endoscopy with negative results, and a full pathophysiologic workup. These strict selection criteria make or result relevant to a particular subgroup of patients, and future studies should be considered in patients from primary and secondary care as well. Finally, as our study was performed just before the new 2017 diagnostic criteria for hEDS was published; therefore, we have used the Brighton Criteria that was the standard method of diagnosing JHS then. It is generally believed that patients given the diagnosis of JHS will meet the new criteria for either hEDS or a related condition described as hypermobile spectrum disorder where patients meet many but all of the criteria for hEDS although this relationship has not been categorically studied. Therefore, further studies are required in FD using the new 2017 hEDS classification criteria to determine the overlap with hEDS and hypermobile spectrum disorder.

Next step forward should be creating more awareness and considering the presence of joint hypermobility as a real and relevant factor in the pathophysiology of FD, and it should, therefore, be studied as such. Consequently, to better understand the involvement of JHS in FD, in all patients, systemic prospective data collection should be contemplated to understand the contribution of connective tissue in pathophysiology of symptoms. These studies might involve both preclinical and clinical studies. For instance, recent studies have shown that extracellular matrix protein TNX is associated exclusively with vagal‐afferent endings and some myenteric neurons in mouse and human stomach, respectively. Furthermore, TNX-deficient mice have accelerated GE and hypersensitivity of gastric vagal mechanoreceptors that can be normalized by an inhibitor of vagal‐afferent sensitivity. Similar studies are required to study the role of collagen and other extracellular matrix proteins in gastrointestinal function.

In conclusion, coexisting JHS is highly prevalent in tertiary care FD patients. No differences were found between FD patients with and without JHS in GE rate, gastric compliance, gastric sensitivity, and GA. No correlations were observed between the Beighton scores and gastric sensorimotor function. Further prospective research will be required to elucidate the relationship between JHS and FD symptoms and pathophysiological features and whether the patients with JHS/FD overlap represent a phenotype that might display differences in heritability, response to treatment, and long-term prognosis from the patients with non-JHS/FD phenotype. Finally, patients with JHS might experience multiple comorbidities such as chronic widespread pain, POTS that can be worsened by meals (16,20,43), and mast cell activation disorder (12,37–40). It is, therefore, important to identify these patients in the gastroenterological clinical practice so that the comorbidities can be recognized as part of the spectrum of disorder that the patient presents with and appropriate referrals are made for their assessment so as to improve their quality of life. Recognition that underlying gastrointestinal symptoms might be due to an underlying heritable connective tissue disorder rather than due to an unknown cause might be helpful in reducing the stigma of experiencing a medically unexplained illness.

## CONFLICTS OF INTEREST

**Guarantor of the article:** Jan Tack, MD, PhD.

**Specific author contributions:** Jan Tack, MD, PhD, and Qasim Aziz, MD, PhD, share senior authorship for this work. J.T., F.C., Q.A., and A.F.: study concept and design. J.T., F.C., and A.F.: acquisition of data. J.T., F.C., and Q.A.: analysis and interpretation of data. J.T., F.C., Q.A., and A.F.: drafting of the manuscript and critical revision of the manuscript. J.T. takes responsibility for the integrity of the work as a whole, from inception to published article. All authors approved the final version of the manuscript.

**Financial support:** Funding was provided by a Methusalem grant from Leuven University to JT.

**Potential competing interests:** None to report.

**Clinical trial registration number:** S56776 (UZ Leuven number reference).

**Study Highlights**

**WHAT IS KNOWN**

✓ There is a strong association between unexplained dyspeptic symptoms and the joint hypermobility syndrome.

**WHAT IS NEW HERE**

✓ Joint hypermobility syndrome is common in patients with functional dyspepsia. Hence, no differences were observed in specific gastric sensory–motor function between patients with and without the joint hypermobility syndrome.

**TRANSLATIONAL IMPACT**

✓ It should be recognized that, at least for a subset of patients with functional dyspepsia, gastrointestinal symptoms may be due to an underlying heritable connective tissue.

## Supplementary Material

SUPPLEMENTARY MATERIAL
