# Supplementary material for: Joint Hypermobility Syndrome in Patients With Functional Dyspepsia
Source: Clin Transl Gastroenterol. 2020 Nov 4;11(11):e00220. doi: 10.14309/ctg.0000000000000220 (PMC7641428; doi:10.14309/ctg.0000000000000220)
Supplement: SUPPLEMENTARY MATERIAL [file ct9-11-e00220-s001.docx]

**SUPPLEMENTARY MATERIAL**

## **JHS assessment**

The patient’s medical history was reviewed for past major illnesses, previous surgeries, relevant hereditary diseases, current illness e.g. diabetes, allergies, current medication use, and the results of standard diagnostic and pathophysiological tests (gastroscopy, GA and GE test) between 2006-2014 during the work-up for the FD diagnosis.

JHS was assessed using a structured interview and examination based on the Brighton criteria for the classification of JHS, which was revised in 1998 (1). These criteria include two major and/or eight minor criteria (see supplementary table 1). The Beighton score, to assess generalized joint laxity (2-4), and the Brighton criteria, however studied in less detail, have previously been accepted as valid and reliable methods to assess JHS (5, 6). Nevertheless, good reproducibility of this assessment can only be achieved after good training and by practicing with a well-structured and standardized assessment which was provided to the investigator doing the clinical assessments (FC) (2, 6, 7).

During the examination, the nine-point examination for Beighton (flexibility) score was performed. One point may be gained for each side for manoeuvres 1 to 4 so that the hypermobility score has a maximum of 9 points if all are positive (table 1). As part of the evaluation, assessment of the presence of scoliosis, Marfanoid habitus and skin hyper-extensibility were carried out.

During the interview, all major and minor criteria of the Brighton classification were assessed. The major criteria included a Beighton score of more than four and arthralgia of four or more joints for longer than 3 months. The minor criteria included a Beighton score of 1 to 3 and arthralgia in 1 to 3 joints or back pain longer than 3 months. Moreover, the occurrence of dislocations/subluxations, three or more soft tissue lesions (e.g. bursitis, tendonitis), abnormal skin (skin striae, hyper-extensibility, abnormal scarring), eyes signs (myopia or dropping eyes), varicose veins, hernias (not gastric hernias) and prolapses (colon, uterine or bladder) were assessed.

JHS was diagnosed by the presence of 2 major, 1 major and two minor or at least four minor criteria (1). The presence of an affected and diagnosed first-degree relative could also be taken into account as a relevant minor criterion. Patients diagnosed with other hypermobility syndromes such as Ehlers–Danlos syndrome type IV, Marfan syndrome or autoimmune joint diseases such as lupus or rheumatoid arthritis, were excluded from this study.

## **Gastric emptying breath test**

The C13-breath test was used to measure gastric emptying rate (8-10). This test is considered standard diagnostic tool for patients with functional dyspepsia at the University Hospital of Leuven in Belgium. After an overnight fast, patients were instructed to ingest a standardized solid meal (breakfast) consisting of one non-radioactive 13C-octanoic acid labelled pancake (244 kcal) within 15 minutes. Before, the meal, two control breath samples are collected. For consumption of the pancake, 5 g sugar was added as a sweetener and 250 ml water was given as a drink. After eating, subjects were requested to provide a breath sample every 15 minutes until 4 hours postprandially. The breath samples were collected in sample tubes and GE rate as analysed by determining the exhaled ^13^CO_2_/ ^12^CO_2_ ratio as previously reported (25-27).

Delayed GE was defined as T½ more than 109 min and rapid GE was defined as T_1/2_ of less than 30 min (11).

## **Gastric barostat study**

A gastric barostat examination had been performed previously as a diagnostic gastric test in the period 2006-2014. Following an overnight fast of at least 12 h, a double lumen polyvinyl tube (Salem sump tube 14 Ch., Sherwood Medical, Petit Rechain, Belgium) with an adherent plastic bag (1200 mL capacity) which was finely folded was introduced through the mouth. The healthy volunteer was then instructed to lie on a specifically designed bed in a sitting position with the knees slightly bent.

The polyvinyl tube was then connected to a computer-driven programmable volume-displacement barostat device (G&J Electronics Inc., Toronto, ON, Canada). To unfold the intragastric bag, it was inflated with a fixed volume of 300 mL of air for 2 min and again deflated completely.

After a 10 min equilibration period, the minimal intragastric distending pressure (MDP) was determined by increasing the intrabag pressure by 1 mmHg every minute until the first stable intrabag volume of 30 mL or more was reached for 2 minutes.

For the evaluation of the gastric sensitivity and compliance stepwise isobaric distensions (MDP+2 mmHg) were initiated. Every 2 minutes the subjects were instructed to rate the intensity of upper abdominal sensation (score: 0: no sensation – 6: epigastric pain or discomfort) induced by each stimulus. The procedure concluded when subjects reported maximal discomfort or pain (score 5-6) or when the intrabag volume reached 1000 mL. For the meal challenge, the pressure level was set at MDP + 2 mmHg. After 30 min baseline period, patients were requested to drink 200 ml of a nutrient liquid meal (Nutridrink®, Nutricia, 150 kcal per 100 ml with 5.9 g proteins, 18.4 g carbohydrates and 5.8 g lipids, Netherlands). Measurement continued for 1 hour and 30 minutes.

For the gastric barostat test, the perception threshold was defined as the first level of pressure and the corresponding volume that evoked a perception score of 1 or more. Discomfort threshold was defined as the first level of pressure and the corresponding volume that provoked a sensation score of five or more. Sensitivity to gastric distention was calculated as the linear slope of the sensitivity curve. Hypersensitivity to distention was defined as the discomfort threshold below 6.6 mm Hg above MDP (12). The gastric compliance of the subjects was calculated as the slope of the volume/pressure curve.

The meal-induced accommodation response was determined as the difference (delta) between the mean volume before (30 min) and after meal intake (60 min). GA was defined as impaired when the delta was found to be smaller than 64 ml (12).

# REFERENCES

1. Fikree A, Aziz Q, Grahame R. Joint hypermobility syndrome. Rheum Dis Clin North Am. 2013;39(2):419-30.

2. Remvig L, Jensen DV, Ward RC. Are diagnostic criteria for general joint hypermobility and benign joint hypermobility syndrome based on reproducible and valid tests? A review of the literature. J Rheumatol. 2007;34(4):798-803.

3. Boyle KL, Witt P, Riegger-Krugh C. Intrarater and Interrater Reliability of the Beighton and Horan Joint Mobility Index. J Athl Train. 2003;38(4):281-5.

4. Smits-Engelsman B, Klerks M, Kirby A. Beighton score: a valid measure for generalized hypermobility in children. J Pediatr. 2011;158(1):119-23, 23.e1-4.

5. Remvig L, Jensen DV, Ward RC. Epidemiology of general joint hypermobility and basis for the proposed criteria for benign joint hypermobility syndrome: review of the literature. J Rheumatol. 2007;34(4):804-9.

6. Remvig L, Engelbert RH, Berglund B, Bulbena A, Byers PH, Grahame R, et al. Need for a consensus on the methods by which to measure joint mobility and the definition of norms for hypermobility that reflect age, gender and ethnic-dependent variation: is revision of criteria for joint hypermobility syndrome and Ehlers-Danlos syndrome hypermobility type indicated? Rheumatology (Oxford). 2011;50(6):1169-71.

7. Juul-Kristensen B, Røgind H, Jensen DV, Remvig L. Inter-examiner reproducibility of tests and criteria for generalized joint hypermobility and benign joint hypermobility syndrome. Rheumatology (Oxford). 2007;46(12):1835-41.

8. Ghoos YF, Maes BD, Geypens BJ, Mys G, Hiele MI, Rutgeerts PJ, et al. Measurement of gastric emptying rate of solids by means of a carbon-labeled octanoic acid breath test. Gastroenterology. 1993;104(6):1640-7.

9. Maes BD, Ghoos YF, Geypens BJ, Mys G, Hiele MI, Rutgeerts PJ, et al. Combined carbon-13-glycine/carbon-14-octanoic acid breath test to monitor gastric emptying rates of liquids and solids. J Nucl Med. 1994;35(5):824-31.

10. Perri F, Clemente R, Festa V, Quitadamo M, Niro G, Andriulli A. 13C-octanoic acid breath test: a reliable tool for measuring gastric emptying. Ital J Gastroenterol Hepatol. 1998;30(2):211-7.

11. Sarnelli G, Caenepeel P, Geypens B, Janssens J, Tack J. Symptoms associated with impaired gastric emptying of solids and liquids in functional dyspepsia. Am J Gastroenterol. 2003;98(4):783-8.

12. Tack J, Caenepeel P, Fischler B, Piessevaux H, Janssens J. Symptoms associated with hypersensitivity to gastric distention in functional dyspepsia. Gastroenterology. 2001;121(3):526-35.
