# Supplementary material for: Joint Hypermobility Syndrome in Patients With Functional Dyspepsia
Source: Clin Transl Gastroenterol. 2020 Nov 4;11(11):e00220. doi: 10.14309/ctg.0000000000000220 (PMC7641428; doi:10.14309/ctg.0000000000000220)
Supplement: SUPPLEMENTARY MATERIAL [file ct9-11-e00220-s002.docx]

**Supplementary Table 1.** **Nine-point Beighton Hypermobility score**

| The Ability to: | Right | Left |
| --- | --- | --- |
| (1) Passively dorsiflex the fifth metacarpophalangeal joint to ≥90° | 1 | 1 |
| (2) Oppose the thumb to the volar aspect of the ipsilateral forearm | 1 | 1 |
| (3) Hyperextend the elbow to ≥10° | 1 | 1 |
| (4) Hyperextend the knee to ≥10° | 1 | 1 |
| (5) Place hands flat on the floor without bending the knees | 1 | |
| TOTAL | **9** | |
